# Drug Amorphous Solid Dispersions Based on Poly(vinyl Alcohol): Evaluating the Effect of Poly(propylene Succinate) as Plasticizer

**DOI:** 10.3390/polym13172922

**Published:** 2021-08-30

**Authors:** Afroditi Kapourani, Artemis Palamidi, Konstantinos N. Kontogiannopoulos, Nikolaos D. Bikiaris, Panagiotis Barmpalexis

**Affiliations:** 1 Department of Pharmaceutical Technology, School of Pharmacy, Aristotle University of Thessaloniki, 54124 Thessaloniki, Greece; akapourag@pharm.auth.gr (A.K.); artemispalamidi@gmail.com (A.P.); kkontogi@cheng.auth.gr (K.N.K.); 2 Laboratory of Polymer Chemistry and Technology, Department of Chemistry, Aristotle University of Thessaloniki, 54124 Thessaloniki, Greece; nbikiaris@gmail.com; 3 Natural Products Research Centre of Excellence-AUTH (NatPro-AUTH), Center for Interdisciplinary Research and Innovation (CIRI-AUTH), 57001 Thessaloniki, Greece

**Keywords:** amorphous solid dispersions, poly(vinyl alcohol), poly(propylene succinate), plasticizer, dronedarone, fusion-based methods

## Abstract

Although significant actions have been taken towards the utilization of poly(vinyl alcohol) (PVA) in the preparation of drug amorphous solid dispersions (ASDs) using fusion-based techniques (such as melt-quench cooling and hot-melt extrusion), several drawbacks regarding its rather high melting temperature and its thermal degradation profile make the use of the polymer extremely challenging. This is especially important when the active pharmaceutical ingredient (API) has a lower melting temperature (than PVA) or when it is thermally labile. In this vein, a previous study showed that newly synthesized polyester-based plasticizers may improve the processability and the thermal properties of PVA. However, the effects of such polyester-based plasticizers on the drug’s physicochemical and pharmaco-technical properties are yet unknown. Hence, the aim of the present study is to extend our previous findings and evaluate the use of poly(propylene succinate) (PPSu, i.e., the most promising plasticizer in regard to PVA) in the preparation of drug-loaded PVA-based ASDs. Dronedarone (DRN), a poorly water-soluble API, was selected as a model drug, and drug ASDs (using either neat PVA or PVA-PPSu) were prepared using the melt-mixing/quench cooling approach at low melting temperatures (i.e., 170 °C). DSC and pXRD analysis showed that a portion of the API remained crystalline in the ASDs prepared only with the use of neat PVA, while the samples having PPSu as a plasticizer were completely amorphous. Further evaluation with ATR-FTIR spectroscopy revealed the formation of significant intermolecular interactions between the API and the PVA-PPSu matrix, which could explain the system’s physical stability during storage. Finally, dissolution studies, conducted under nonsink conditions, revealed that the use of PVA-PPSu is able to maintain DRN’s sustained supersaturation for up to 8 h.

## 1. Introduction

Poly(vinyl alcohol) (PVA) is a semicrystalline, nonionic, water-soluble polymer, available in a range of grades with various degrees of hydrolyzation and molecular weights (MWs) [1,2,3,4,5]. Its hydrolysis levels (i.e., the ratio of hydrophilic polyvinyl alcohol to hydrophobic polyvinyl acetate groups) significantly affect the various physicochemical and thermomechanical properties of the polymer, such as the aqueous solubility, swelling ability, flexibility and tensile strength [4,6,7]. The highly tunable unique features of PVA, including its low toxicity [8,9,10], noncarcinogenicity and excellent biodegradability [11,12], make it an appealing matrix-carrier for a variety of medicinal and pharmaceutical applications. This is the reason why in the last few years, PVA has gained the attention of several pharmaceutical formulation scientists for the enhancement of drugs’ poor aqueous solubility via the preparation of amorphous solid dispersions (ASDs) based on fusion-based pharmaceutical formulation processes [13,14].

ASDs are defined as dispersions of one or more active pharmaceutical ingredients (APIs) in an inert matrix-carrier (either polymeric or not) at a solid state prepared by several methods, such as fusion, solvent and grinding [15,16,17,18,19,20,21]. Amongst the several preparation methods, fusion-based techniques, such as melt-quench cooling, hot-melt extrusion (HME) and KinetiSol (a fusion-based process that utilizes frictional and shear energies to rapidly transition drug–polymer blends into a molten state), are considered to be more advantageous since they are more easily scalable, solvent-free, continuous, fast and robust [22,23,24,25,26]. Therefore, within this set framework, several excipient manufacturers have focused their product pipeline on the development of specifically designed PVA grades suitable for the preparation of fusion-based drug ASDs, such as Merk’s Parteck MXP, which is a specially designed PVA grade suitable for HME-based ASDs [27].

However, and despite this wide interest, the use of PVA as a sole matrix-carrier in fusion-based pharmaceutical formulations is considered to be extremely challenging, because the polymer is not extrudable at temperatures below its melting point (~200 °C), while it is regarded as a rather thermally sensitive compound with thermal degradation occurring near its melting point (~250 °C) [4,11,28]. These drawbacks may significantly limit the number of APIs suitable for PVA-based fusion processing, especially in the cases where the formulated drug has a rather low melting point (as compared to PVA) or is thermally unstable.

In an attempt to improve PVA’s thermal processability and hence overcome the aforementioned drawbacks, plasticizers may be added. In these cases, i.e., when a plasticizer is added, a large reduction in frictional forces between the polymer chains is responsible for the lowering of the extrusion temperature, improving melt flow during processing (such as in the case of hot-melt extrusion) and improving the characteristics of the final resulting product (for example a filament) by reducing its brittleness [29,30]. Within the same concept, a recent publication has evaluated the use of poly(alkylene succinate)-based oligomers as PVA plasticizers suitable for fusion-based pharmaceutical applications [31]. Results in this study showed that the utilization of low molecular weight (MW) poly(propylene succinate) (PPSu) was able to reduce the processing temperature of PVA and improve its melt flow properties (estimated via melt flow index measurement) without affecting its thermal decomposition.

Therefore, with the present study, we attempted to expand our previous findings by evaluating the use of the most promising poly(alkylene succinate) plasticizer (i.e., PPSu) in the preparation of PVA-based drug ASDs. Specifically, in the present study, drug ASDs were prepared, via melt-mixing/quench cooling approach, using PVA as a matrix-carrier and PPSu as a plasticizer. Dronedarone (DRN), an antiarrhythmic drug, structurally related to amiodarone [32,33,34], was used as a suitable model drug, since it is a poorly water-soluble API (practically insoluble in water, i.e., 0.64 mg/mL [35]), while it melts at a rather low melting temperature (i.e., ~140 °C), which makes it an ideal candidate for the evaluation of PVA’s processability at low temperatures (i.e., below 200 °C). The effect of the plasticizer within the prepared ASD system was evaluated in terms of processability and in regard to its effect on several physicochemical, thermophysical and pharmaco-technical characteristics of the prepared ASDs.

## 2. Materials and Methods

### 2.1. Materials

**Figure 1:**
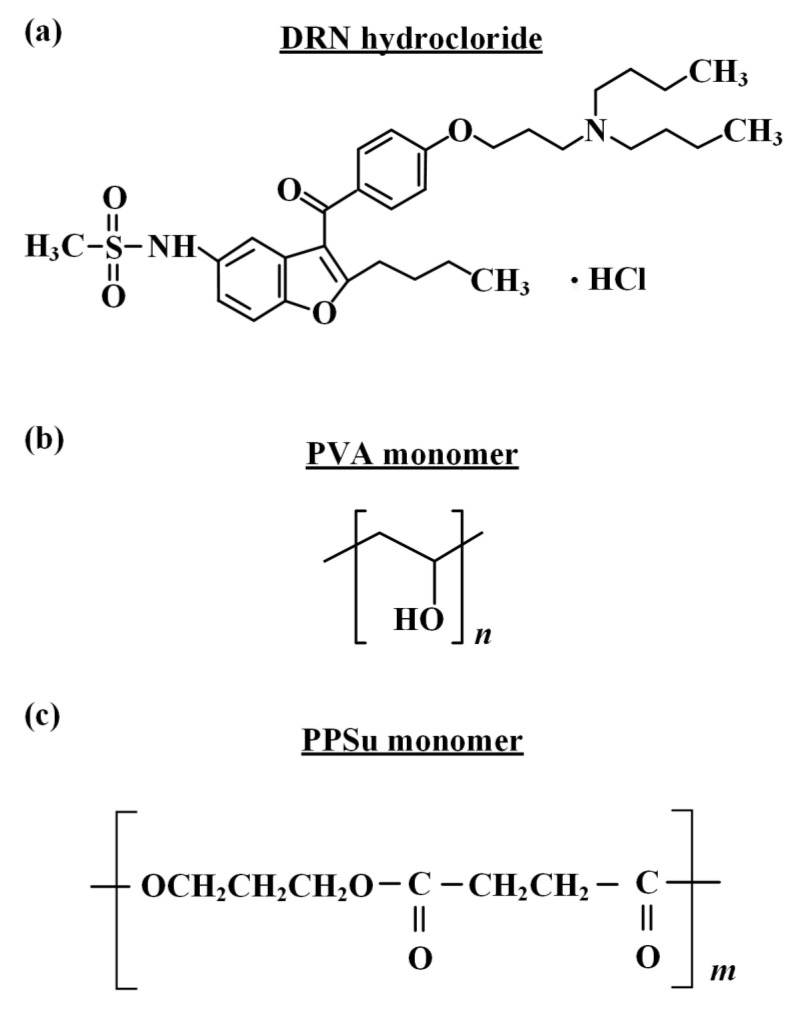
Chemical structures of (**a**) DRN (HCl salt), (**b**) PVA monomer and (**c**) PPSu monomer.

Dronedarone (DRN, as a hydrochloride salt, Figure 1) with purity over 99.5% *w*/*w*, molecular weight (Mw) of 556.756 and logP 4.63, was kindly given as a gift from Rontis Hellas S.A. (Athens, Greece). Partially hydrolyzed PVA (Parteck MXP, lot No. F2016164812), shown in Figure 1, with 87–89% hydrolysis grade and MW approx. 32,000 Da, was obtained from Merck Millipore (Merck Millipore, Burlington, MA, USA). Succinic acid (SA) (purum 99%+) and ethylene glycol (anhydrous, 99.8%), used in the synthesis of the PPSu plasticizer, were purchased from Sigma-Aldrich Chemical Co. (St. Louis, MO, USA); 1,3-propanediol (purum 99.6%+) and 1,4-butanediol (assay 99%) were purchased from Alfa Aeser, (Kandel, Germany). Antimony trioxide (Sb_2_O_3_, 99.99%), used as catalyst, was of analytical grade and was purchased from Aldrich Co. (Chemie GmbH, Steinheim, Germany). All other reagents were of analytical or pharmaceutical grade and used as received.

### 2.2. Preparation of PPSu

The preparation of the plasticizer (i.e., PPSu, Figure 1) was made via a two-stage melt polycondensation method (esterification and polycondensation) in a glass batch reactor based on a previously published methodology [31]. Briefly, accurately weighted amounts of succinic acid and diol (at a molar ratio of 1/1.1) were placed into a round bottom flask, along with the catalyst Sb_2_O_3_ (400 ppm relative to succinic acid). The reagents were evacuated several times and filled with nitrogen (N_2_) in order to remove the oxygen. During the first reaction step (esterification), the reagents were heated at 180 °C for 4 h under 500 rpm stirring and N_2_ flow (50 mL/min). Water was distilled (as reaction by-product) and was collected in a graduated cylinder. When almost all the theoretical amount of H_2_O was collected (after 3.5 h), the first step was considered as completed. Thereafter, in the second step of polycondensation, temperature was gradually increased from 180 to 230 °C (15 min) and vacuum was slowly implemented (5.0 Pa) in order to remove unreacted monomers, avoid excessive foaming and minimize oligomer sublimation. The polycondensation continued for about 15 min, at 230 °C, while stirring speed was also increased to 750 rpm. After that time, polymerization was terminated by rapid cooling to room temperature and the polyester was removed. The prepared PPSu polyester was stored in hermetically sealed glass vials and placed in desiccators (25 °C) before further use.

### 2.3. Preparation of ASDs

Accurately preweighed amounts (total of 10 g) of either neat PVA-PPSu (in ratios of 90/10, 80/20 and 70/30% *w*/*w* of PVA to plasticizer) or PVA-PPSu/DRN (in ratios of 90/10, 80/20 and 70/30% *w*/*w* of matrix-carrier to API) were thoroughly mixed using a mortar and pestle, and then melt solid dispersions were prepared via melt-mixing in a Haake-Buchler Reomixer (Model 600, Haake Technik GmbH, Vreden, Germany) with roller blades. Before melt-mixing, all prepared blends were thoroughly dried in an oven at 40 °C for 24 h. Melt-mixing was performed at 170 °C for either 3 or 10 min with a 30 rpm roller speed. All solid dispersions after preparation were quench-cooled and ground in a freezer mill (Horiba 6770, Horiba Scientific, Kyoto, Japan). In addition to PVA-PPSu/DRN, PVA/DRN ASDs (i.e., without the use of the plasticizer) were also prepared for comparison. All samples after preparation were stored in hermetically closed glass containers (a rubber stopper reinforced with an Apiezon Q seal was used) and placed in zero-humidity desiccants (using P_2_O_5_) before further use.

### 2.4. Hot Stage Polarized Microscopy (HSM)

The effect of PPSu on the melting behavior of the binary (PVA/DRN) and ternary (PVA-PPSu/DRN) systems was evaluated via HSM. Specifically, physical mixtures of PVA-PPSu/DRN and PVA/DRN at a ratio of 30/70 of API to matrix-carrier were heated from 25 °C until complete melting (at a rate of 10 °C/min) on a Linkam THMS600 heating stage (Linkam Scientific Instruments Ltd., Surrey, UK) mounted on Olympus BX41 polarized light microscope and controlled through a Linkam TP94 temperature controller. Evaluation of the system’s melting point was made by visual observation.

### 2.5. Differential Scanning Calorimetry (DSC)

The thermal properties of all samples (i.e., the neat PVA/PPSu matrices and the drug ASDs) were evaluated in a DSC 204 F1 Phoenix heat flux instrument (NETZSCH, Germany). Specifically, accurately weighted quantities (~5.0 mg) of samples were melt-quenched in situ by initially heating them in the DSC pan at 220 °C (with a heating rate of 10 °C/min); keeping in isothermal condition for 3 min, in order to erase any thermal history; cooling at a rate of 20 °C/min to approximately 20 °C; and then reheating again at a heating rate of 10 °C/min up to 210 °C. All melting points were determined as the onset temperature of the recorded endothermic heat flow curve (T_m,onset_); glass transition temperature (T_g_) was determined at the inflection point temperature, while enthalpy of fusion (ΔH_f_) was determined as the integrated area of the heat flow curve in all cases. The standard deviations of temperatures and enthalpies determined in this work were not higher than 1.0 °C and 3.0 J/g, respectively. Nitrogen flow (50 mL/min) was applied in order to provide a constant thermal blanket within the DSC cell. The instrument was calibrated for temperature using high purity benzophenone, indium and tin, while the enthalpic response was calibrated using indium. Thermograms were analyzed using the NETZSCH Proteus Thermal Analysis software package version 5.2.1 (NETZSCH, Germany). All experiments were conducted in triplicate.

### 2.6. Thermogravimetric Analysis (TGA)

Thermogravimetric analysis (TGA, Shimadzu TGA-50 thermogravimetric analyzer, Tokyo, Japan) was employed in order to evaluate components’ thermal stability during ASD preparation process. For the analysis, approximately 10.0 mg of samples were placed into suitable aluminum sample pans, attached to a sensitive microbalance assembly and heated from 30 to 300 °C at a rate of 10 °C/min, using nitrogen as purge gas at a flow rate of 50 mL/min. The weight variation of the samples was recorded in relation to temperature, while all experiments were conducted in triplicate.

### 2.7. Powder X-ray Diffractometry (pXRD)

pXRD patterns of the pure components, PVA-PPSu and PVA-PPSu/DRN ASDs (prepared according to Section 2.3) were recorded using an X-ray diffractometer (Rigaku Miniflex II) with a CuKα radiation source for crystalline phase identification (λ = 0.15405 nm for CuKα) immediately after preparation. All samples were scanned from 5 to 45°.

### 2.8. Attenuated Total Reflection Fourier Transform Infrared (ATR-FTIR) Spectroscopy

Possible molecular interactions among the API and the two matrix components (i.e., PVA and PPSu) were experimentally evaluated via ATR-FTIR spectroscopy. Specifically, the ATR-FTIR spectra of the raw materials (DRN, PVA and PPSu), as well as all ASDs, were collected in the region of 4000–750 cm^–1^ using a Shimadzu IRPrestige-21 FT-IR spectrometer (Tokyo, Japan), coupled with a horizontal Golden Gate MKII single-reflection ATR system (Specac, Kent, UK) equipped with ZnSe lenses after appropriate background subtraction. Sixty-four scans over the selected wavenumber range with a spectral resolution of 4 cm^−1^ were averaged for each sample.

### 2.9. Supersaturation Dissolution Studies

Supersaturated dissolution studies of the prepared ASDs, as well as the neat amorphous DRN, were carried out in an apparatus II (paddles) dissolution tester (PT-DT7 Pharma Test AG, Hainburg, Germany), using 100 mL of hydrochloric acid buffer (pH 1.2). The temperature of the dissolution medium was maintained at 37 °C ± 0.5 °C, and the paddle rotation was set at 100 rpm. Nonsink conditions were maintained during the dissolution study in order to build up the supersaturation that commonly occurs under finite volume conditions in the gastrointestinal (GI) tract and results in the associated nucleation and crystallization events. [36]. The degree of departure from sink condition was characterized by a dimensionless sink index (*SI*), which is defined as the ratio of the product of *CsV* to *Dose*, where *Cs* is the solubility of the crystalline drug, *V* is the volume of dissolution medium and *Dose* is the total amount of drug in the sample. In this study, the dissolution profiles of DRN from the melt mixings were tested under extremely nonsink conditions (i.e., *SI* = 0.15). Generally, a larger *SI* corresponds to a dissolution system that is closer to the sink condition (the perfect sink condition is achieved when *SI* > 10). During the dissolution trials, at predetermined time intervals (5, 10, 15, 30, 45, 60, 90, 120, 180, 240, 300, 360, 420 and 480 min), 3.0 mL of aliquot was removed and replaced with 3.0 mL of fresh dissolution medium. The aliquot was instantly passed through a 0.1 μm Whatman filter and diluted with MeOH in order to eliminate the risk of drug crystallization after sampling. The drug concentration was determined spectrophotometrically using a UV-Vis spectrometer (Pharma Spec UV-1700, Shimadzu Europa GmbH, Duisburg, Germany) at 293 nm. Each test was performed in triplicate.

### 2.10. Stability Studies

ASD formulations were stored in open vials under International Council for Harmonization of Technical Requirements for Pharmaceuticals for Human Use (ICH) long-term storage conditions (25 ± 2 °C/40 ± 5% RH). After three months of storage, all samples were studied by pXRD to assess the drug’s structural changes, using the same methodology and conditions described in Section 2.7.

## 3. Results and Discussion

### 3.1. Evaluation of Melt-Mixing Time’s Effect on the Neat PVA-PPSu Matrices

Initially, and before proceeding with the evaluation of the drug-loaded ASDs, it is important to investigate the effect of the melt-mixing time on the neat polymeric PVA/PPSu matrices’ characteristics.

**Figure 2:**
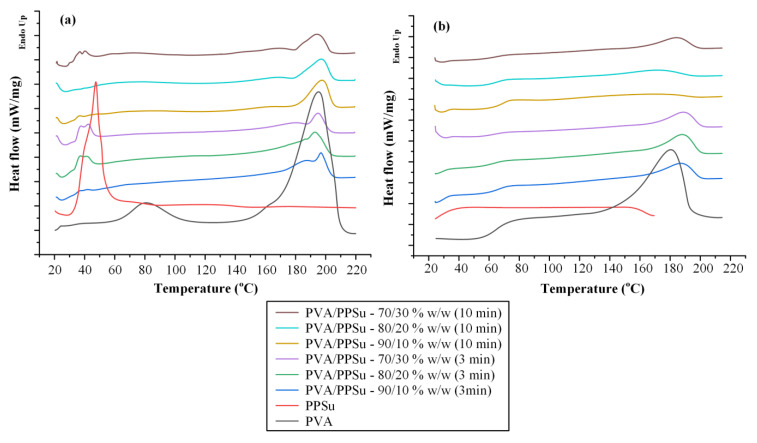
DSC thermograms after the 1st (**a**) and the 2nd (**b**) heating scans for the neat PVA/PPSu matrices.

Figure 2 depicts the DSC thermograms (both first and second heating scans) of the neat PVA-PPSu matrices prepared after 3 and 10 min of mixing, while Table 1 summarizes all the recorded thermal events.

**Figure 3:**
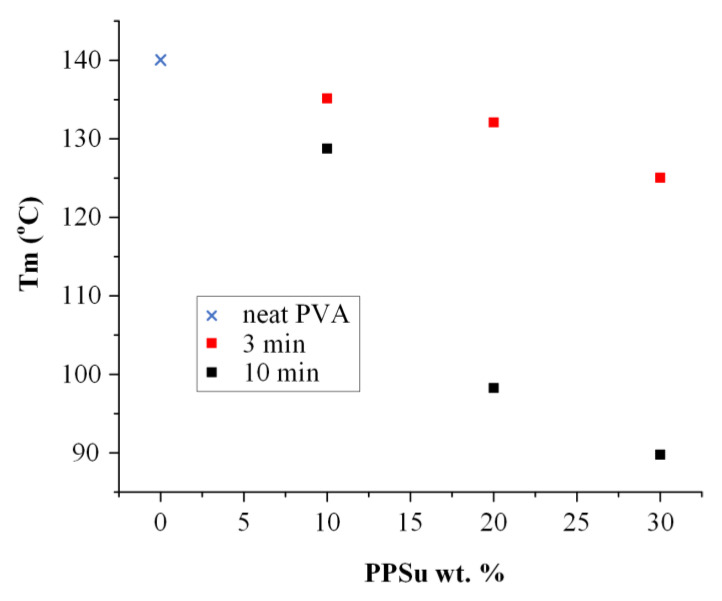
PVA’s Tm vs. PPSu % weight content for the neat PVA/PPSu matrices prepared after 3 or 10 min of melt mixing.

As far as the neat PVA is concerned, an initial broad endotherm peak (starting from ~70 °C and extending up to ~105 °C) was recorded during the first heating scan, which is related to the absorbed moisture water, followed by a second endothermic peak (starting at 155.1 °C, ΔH_f_ = 42.5 J/g), corresponding to the melting of the neat polymer. Upon the second heating scan, DSC thermograms for the neat PVA showed a T_g_ value at 68.4 °C, followed by a broad endothermic peak with T_m,onset_ at 140.1 °C (ΔH_f_ = 30.8 J/g). With respect to the neat PSSu, DSC thermograms from the first heating scan showed a single endothermic melting peak at 45.3 °C (ΔH_f_ = 51.9 J/g), while no thermal events were recorded during the reheating, at least in the evaluated temperature range. Looking at the thermograms obtained for the neat PVA/PPSu matrices after the first heating scan, results showed clearly that the increase in mixing time led to reduced PPSu crystallinity. Specifically, independently of the plasticizer content, in all cases, the endothermic peak corresponding to the melting of PPSu was significantly reduced in the samples prepared after 10 min of mixing, as compared to 3 min (this is also depicted from the ΔH_f_ values recorded in Table 1). Similarly, as in the case of the plasticizer, the crystallinity of PVA also decreased when the mixing time was raised, irrespectively of PPSu content. Focusing now on the main aspect of the present study (i.e., the plasticizing effect of PPSu on PVA), Figure 3 shows the recorded variations of polymer’s T_m,onset_ with respect to plasticizer content for several melt-mixing times. Results showed that in all cases (i.e., irrespectively of the mixing time) the melting onset of the polymer was significantly decreased in the presence of PPSu. These findings suggest that the selected polyester (at least at the weight content investigated) can be considered as a good plasticizer for PVA. In addition, what is more interesting to mark is that, according to the obtained results, it appears that an interplay takes place between the content of the plasticizer and the melt-mixing time. Specifically, results from Figure 3 clearly show that as the PPSu content increases (i.e., above 10 wt.%), a further reduction in PVA’s melting onset is obtained for the matrices prepared after 10 min of mixing, indicating that the utilization of proper melt-mixing conditions (i.e., an increase in mixing time) can further assist the plasticizing effect of the selected low-MW polyester. At this point, it should be noted that a similar plasticizing effect on PVA’s thermal properties has been also proved for other components, such as mannitol, sorbitol, urea and glycerol [30]. However, this does not suffice to regard them as successful plasticizers, since it is also vital to ensure the acceptable thermal processability, miscibility and stability of the PVA–plasticizer system. For example, conventional water-soluble plasticizers for PVA, such as glycerol, induce moisture absorption that might negatively affect the performance of PVA materials during their applications [12].

**Figure 4:**
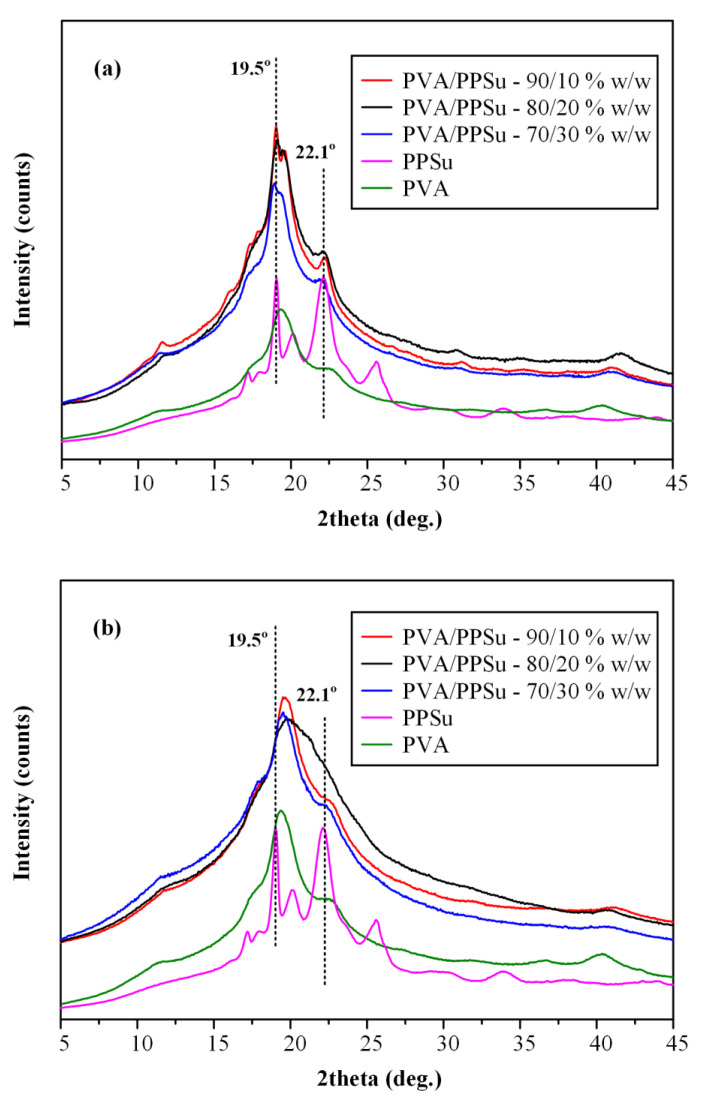
pXRD diffractograms of the raw materials (PVA and PPSu), as well as the neat PVA/PPSu matrices prepared after 3 min (**a**) and 10 min (**b**) of melt-mixing.

In addition to the thermal properties, the effect of the melt-mixing time on the physical state of the neat polymeric matrices was also evaluated. Figure 4 illustrates the pXRD diffractograms of the raw materials (PVA and PPSu), as well as the neat PVA-PPSu matrices prepared after 3 and 10 min of melt-mixing.

Neat PVA’s pXRD diffractogram showed an amorphous halo along with a characteristic strong crystalline reflection at 2θ of ~19.5° accompanied by a small shoulder at ~22.6°. Similarly, PPSu revealed an amorphous halo with four main diffractogram peaks at 19.1, 20.2, 22.1 and 25.6°, respectively. Hence, based on the obtained results, it can be said that both raw materials were initially (i.e., before the melt-mixing process) semi-crystalline in nature. Moving on with the diffractograms collected for the neat PVA-PPSu, it is obvious that the matrices prepared after only 3 min of melt-mixing remained semi-crystalline in nature, with the characteristic peaks of PPSu (at 2θ of 19.5 and 22.1°) clearly shown in all tested PVA/PPSu ratios, while in the matrices prepared after a longer mixing time (i.e., 10 min) a significant reduction in the crystallinity was observed, since all pXRD characteristic diffractogram peaks corresponding to the plasticizer (i.e., PPSu) were missing. Therefore, based on the obtained results, it can be said that the increase in melt-mixing time results in a significant reduction in PVA/PPSu crystallinity, a feature that is extremely important when designing drug ASDs, as the presence of crystals in the polymeric matrix can be the substrate for API’s recrystallization (and therefore physical instability) during storage.

**Figure 5:**
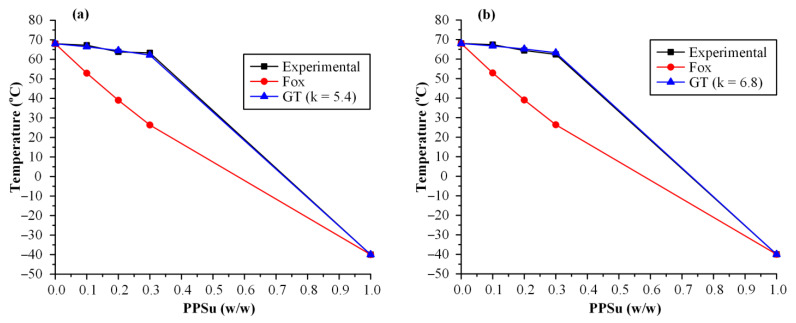
Prediction of T_g_−composition dependence in PVA−PPSu blends prepared after 3 min (**a**) or 10 min (**b**) of melt-mixing, using either the Fox or the Gordon–Taylor (GT) fitting equations.

In a further attempt to study the reasons behind this reduction in PVA/PPSu crystallinity, the effect of melt-mixing time on the formation of molecular interactions between the two components was initially evaluated via DSC. Generally, there are various theoretical and empirical formulae that describe the dependence of the T_g_ on the mass fractions and T_g_ values of the initial components in order to evaluate the possible molecular interactions. Amongst them, the Fox model described by the following equation is widely used [37]:

[1/T_g_] = w_1_/T_g1_ + w_2_/T_g2_ (1)

where T_g_ is the glass transition temperature of the polymer/plasticizer blend; w_1_ and w_2_ are the mass fractions of the PVA and PPSu, respectively, and T_g1_ and T_g2_ are their respective glass transition temperatures. Similarly, Gordon and Taylor (GT) have proposed another equation considering the possibility of significant interactions evolving between the studied components [37]:

T_g_ = (w_1_T_g1_ + kw_2_T_g2_)/(w_1_ + kw_2_) (2)

where k is a is a curve fitting parameter representing the semiquantitative measure of the interaction strength between the reactive groups. During data fitting, the k value having the best (higher) correlation coefficient value is chosen in each case. If k takes values above 1, then strong interactions between the blend components are taking place [38,39]. It is important to note (before proceeding with the analysis of the results) that the miscibility of the two components and the presence of a single T_g_ for the two components (both are important prerequisites needed in order to use the Fox and GT models) were verified in a previous study [31]. Figure 5 shows the experimentally determined T_g_ values along with the theoretical predictions derived from either the Fox or the GT approach for the neat PVA-PPSu matrices.

**Figure 6:**
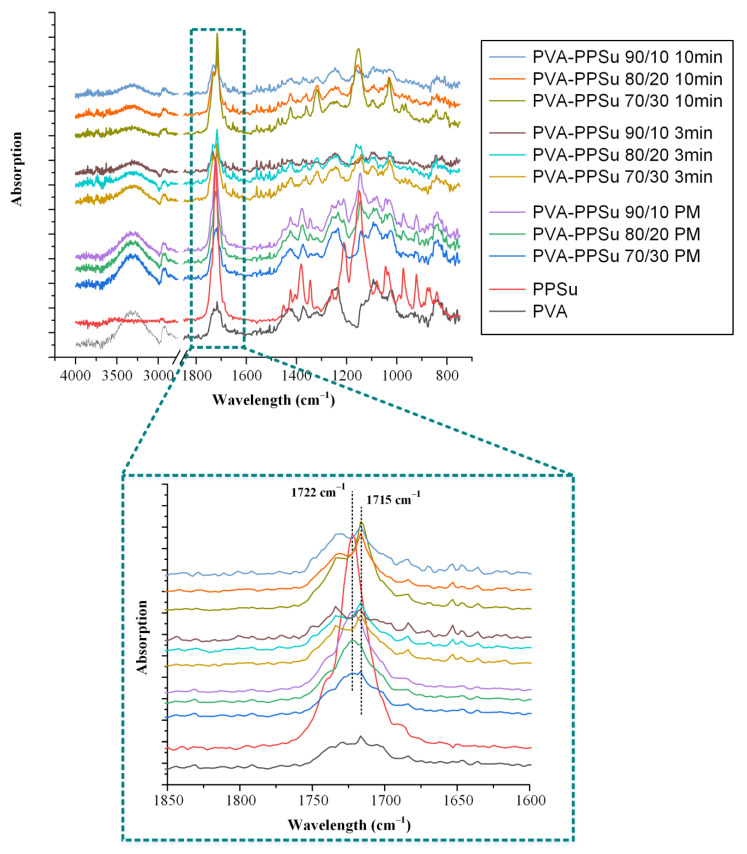
ATR−FTIR spectra of raw materials (PVA and PPSu), their physical mixtures (PMs) and melt−fusion dispersions prepared after 3 and 10 min of melt-mixing.

According to the obtained results, in contrast to the Fox fitting results, where the theoretically estimated T_g_ values were not in good agreement with the recorded experimental values, the use of GT showed excellent fitting with k parameter being well above unity (5.4 and 6.8 for matrices prepared at 3 and 10 min of melt-mixing, respectively) indicating that important molecular interactions are evolving between the polymer and the plasticizer. The evaluation of the type and the strength of these molecular interactions evolving between the two matrix components (i.e., polymer and plasticizer) was made by ATR-FTIR spectroscopy. Specifically, Figure 6 shows the ATR-FTIR spectra of the raw materials (PVA and PPSu), their physical mixtures (PMs, prepared in a mortar and pestle) and their melt dispersions prepared after 3 and 10 min of melt-mixing. In regard to the raw material, the ATR-FTIR spectrum of PVA revealed several characteristic peaks at 3300 cm^−1^ due to the stretching vibrations of -OH, at 1726 cm^−1^ due to the stretching of the -C=O from the remaining acetyl groups, at 1421 cm^−1^ due to the bending of -OH and the wagging of -CH_2_ groups, at 1323 cm^−1^ due to the δ(OH) rocking and the CH wagging, at 1146 cm^−1^ which can be ascribed to the stretching of the -CO- group from the crystalline part of polymer and at 1085 cm^−1^ due to the bending of the -OH group from the amorphous part of PVA. Similarly, PPSu also showed several characteristic ATR-FTIR peaks with the more pronounced located at 1722 and 1158 cm^−1^, assigned to the -C=O and -COO- asymmetric stretching vibrations, respectively. Regarding the polymer–plasticizer PMs, results showed that, in all cases, the recorded ATR-FTIR spectra were the sum of the individual components, and hence no molecular interactions are evolving between the two components during their physical mixing. On the contrary, the spectra of the melt dispersions showed several differences as compared to the respective PMs. Based on the obtained results, there is a clear reduction and a widening of the PVA peak recorded at 3300 cm^−1^ (corresponding to the -OH stretching of PVA) while a small shift (from 1722 to 1715 cm^−1^) is also recorded for the -C=O and -COOC- stretching of the PPSu plasticizer. Similar results have also been reported from a previous study where the use of PPSu as PVA’s plasticizer, suitable for fusion-based pharmaceutical applications, was evaluated for the first time [31]. These differences were more pronounced in the case of PVA-PPSu matrices prepared at 10 min of melt-mixing, as compared to those prepared for only 3 min, indicating that stronger molecular interactions, probably in the form of hydrogen bonds (HBs) between the hydroxyl hydrogens of PVA and the ester oxygens of the plasticizer, are being formed in the matrices prepared with increasing melt-mixing time.

Therefore, based on the above analyses, it can be concluded that the use of 10 min of melt-mixing favors the formation of a more homogeneous amorphous PVA-PPSu matrix (which is more suitable for the preparation of drug ASDs), and hence, 10 min of melt-mixing was selected for the preparation of all drug-loaded ASD samples.

### 3.2. DRN-Loaded PVA-PPSu ASDs

In the following section, the effect of the plasticizer (set at a constant weight ratio of 30 wt.%) on the preparation of PVA-based drug-loaded ASDs will be thoroughly evaluated. In all cases, along with the PVA-PPSu/DRN ASDs, similar drug ASD formulations having only the polymer as a matrix-carrier (i.e., without the addition of the plasticizer) were prepared and evaluated for comparison.

#### 3.2.1. Thermal Stability via TGA

**Figure 7:**
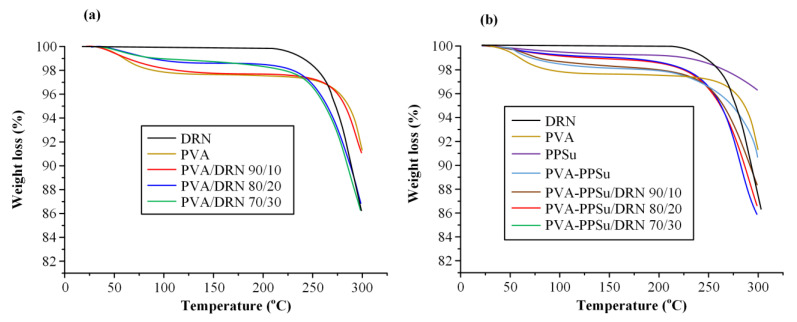
TGA thermograms of all raw materials along with PVA/DRN (**a**) and PVA-PPSu/DRN (**b**) samples at several weight ratios.

Before proceeding with the analysis of the drug-loaded ASDs it is important to verify that all components were stable during their thermal treatment. Figure 7 presents the TGA thermograms for all raw materials (i.e., DRN, PVA and PPSu) along with the respective neat PVA-PPSu matrices and the PVA/DRN and PVA-PPSu/DRN samples at various weight ratios.

Results showed that DRN is thermally stable until ~220 °C, while PVA, which showed an initial small weight loss (~2 wt.%) due to the presence of residual moisture water, was stable when heated up to ~250 °C, and PPSu was stable when heated up to ~220 °C. In the case of the neat PVA-PPSu matrices and the PVA/DRN or PVA-PPSu/DRN formulations, results from the TGA graphs showed good thermal stability (up to ~ 210 °C) irrespectively of the drug content, indicating that the selected melt-mixing temperature (i.e., 170 °C) was adequate for the preparation of the drug ASDs.

#### 3.2.2. Physical State Evaluation via pXRD

**Figure 8:**
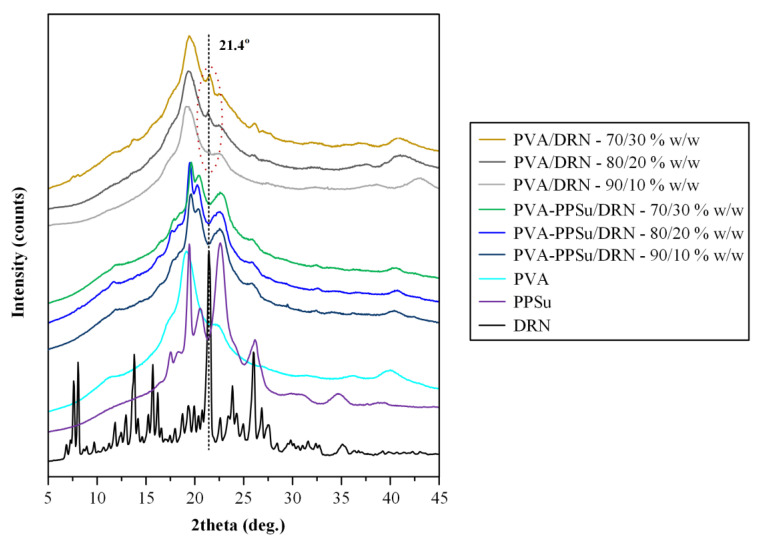
pXRD diffractograms of all raw materials along with PVA/DRN and PVA-PPSu/DRN ASDs at several weight ratios.

Figure 8 illustrates the pXRD diffractograms of the neat DRN and the ASDs prepared at several weight ratios. The pXRD pattern of DRN showed several peaks with higher intensities at 2θ of 7.6, 8.1, 13.8, 15.7, 16.3, 21.4, 23.8 and 26.0°. The obtained diffractogram was similar to the patterns published previously and corresponds to the form I polymorph of the API [35,40]. Looking at the results obtained for the PVA-PPSu/DRN ASDs, it is obvious that the characteristic peaks of DRN were completely missing in all recorded diffractograms, indicating that the drug was amorphously dispersed within the selected polymer/plasticizer matrix. On the contrary, the patterns recorded for all PVA/DRN ASD samples revealed a characteristic DRN diffractogram peak at 2θ of 21.4° (corresponding to form I crystals) indicating that at least a portion of the API remained crystalline when only PVA was used. Based on these findings, it seems that the addition of PPSu and its plasticizing effect improves the miscibility of the API within the PVA matrix when low processing temperatures are being used, resulting in a more homogeneous molecular dispersion of the API within the prepared matrix, as compared to the ASDs using only the neat PVA where, due to its poor thermophysical properties, a portion of the API either remained crystalline during the melt-mixing process, or it was not homogeneously dispersed within the molten parts of the polymer (i.e., poor miscibility), resulting in its recrystallization during quench-cooling and short storage (i.e., few hours until its physical state evaluation).

#### 3.2.3. Evaluation of Thermal Properties

**Figure 9:**
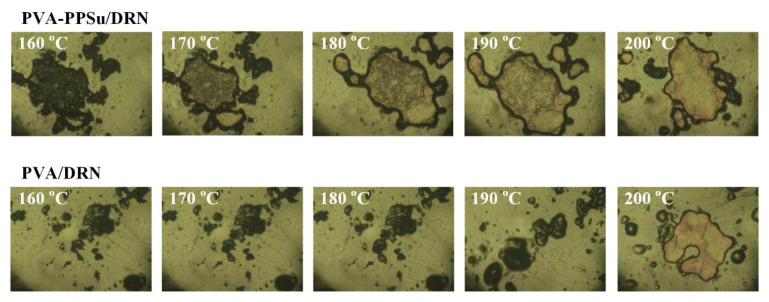
HSM images during melting of the PMs of PVA-PPSu/DRN and PVA/DRN (at a matrix-carrier to API ratio of 30/70% *w*/*w*).

In order to evaluate the previous hypothesis regarding the plasticizing effect of PPSu in the preparation of PVA-based drug-loaded ASDs, the melting behavior of the PMs of the binary (PVA/DRN) and ternary (PVA-PPSu/DRN) samples was initially evaluated via HSM. Results in Figure 9 showed that with the addition of the PPSu, the melting of the drug-loaded PVA PMs starts at approximately 30 °C lower as compared to the samples having only the polymer (i.e., 170 and 200 °C, respectively). Hence, it can be said that the use of the PPSu polyester clearly improves the melt-processing performance of PVA at temperatures lower than its melting point.

**Figure 10:**
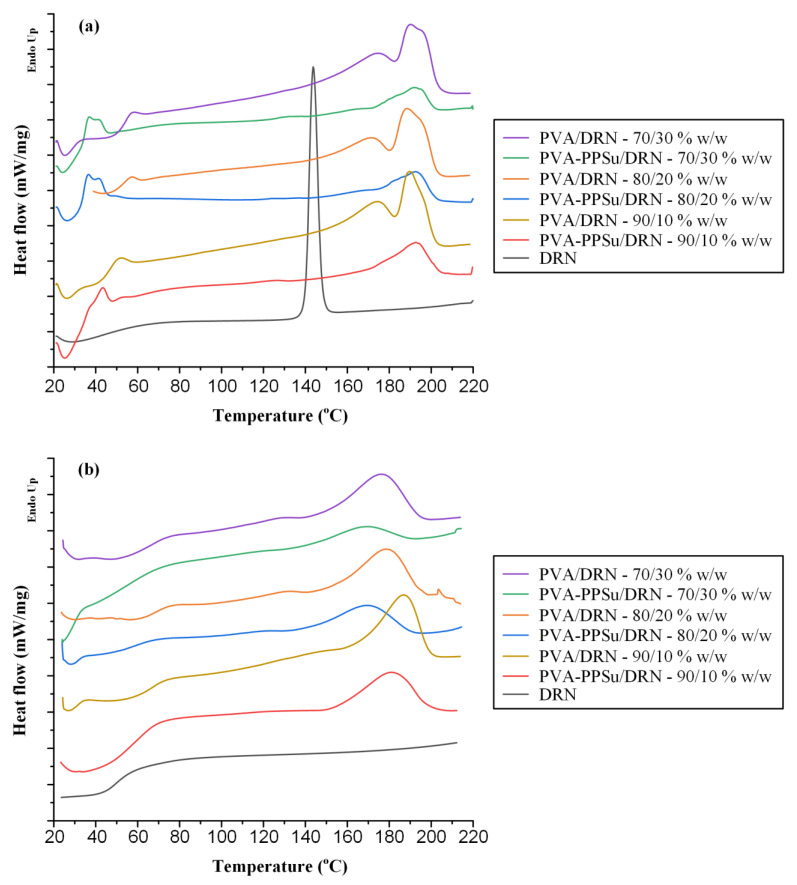
DSC thermograms after the 1st (**a**) and the 2nd (**b**) heating scans for the DRN-loaded ASDs.

In addition to HSM, the thermal properties of the prepared ASDs were also evaluated via DSC. Figure 10 shows the DSC thermograms (both first and second heating scans) of all prepared ASDs using either PVA or PVA-PPSu as matrix-carriers, while Table 2 summarizes all the recorded thermal events.

In the case of the neat DRN, results from the first heating scan showed a single endotherm (peak onset at 141.6 °C and max at 143.7 °C) corresponding to the melting of the DRN form I crystals, while upon reheating only a single T_g_ was recorded at 49.5 °C. Therefore, according to its thermal characteristics, the API can be characterized as a stable glass-former, since it does not recrystallize after reheating (i.e., it is a class III glass forming ability (GFA) compound according to the classification system of Baird et al. [41]). Regarding the first heating scan of the PVA-PPSu/DRN ASDs, all recorded thermograms showed two melting endotherms, the first corresponding to the plasticizer (PPSu) and the second to the polymer (PVA), with no traces of DRN’s melting, indicating that the API was amorphously dispersed within the matrix-carrier. On the contrary, the DSC thermograms of the ASDs that did not contain the plasticizer (i.e., PVA/DRN formulations) revealed traces of DRN’s melting (onset at ~142 °C) in all weight ratios evaluated, indicating that, at least when low melting temperatures are used for the preparation of PVA-based ASDs, the API is not fully amorphized when the plasticizer is not present. Finally, the plasticizing effect of PPSu is also shown in the second DSC heating scan results, where the T_g_ values of the ASDs prepared with the addition of the plasticizer were approximately 10 °C lower than those prepared with the neat PVA (keeping in mind that only a single T_g_ was recorded in all cases).

#### 3.2.4. Evaluation of Molecular Interactions

In any ASD system, the preparation and performance (in terms of physical stability and release) of the resultant formulations is highly dependent on the type and strength of the intermolecular interactions evolving between the API and the matrix-carrier(s). Generally, weak physical bonds formed by noncovalent interactions (such as HBs, ionic bonds, van der Waals, dipole–dipole interactions and acid–base interactions) are common interactions occurring between the API and the rest of the ASD components, with the HB formation being the most frequently noticed [42,43,44,45,46]. The establishment of these intermolecular interactions usually prevents the API molecules from self-association and, consequently, recrystallization. Therefore, in the present study, the possible formation of intermolecular interactions between the API and the matrix-carrier was evaluated via ATR-FTIR spectroscopy.

**Figure 11:**
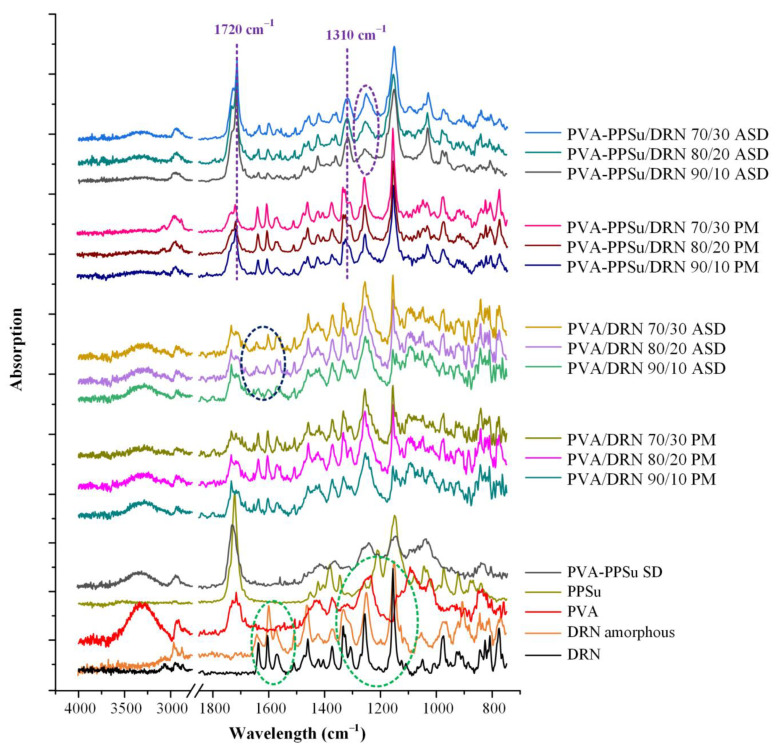
ATR−FTIR spectra of the initial raw materials, the pure amorphous DRN, the neat PVA−PPSu matrix after melt-quench cooling (PVA−PPSu SD) and the binary (PVA/DRN) and ternary (PVA−PPSu/DRN) ASDs with their PMs at several weight ratios (green dashed circles highlight the differences between the amorphous and the crystalline DRN spectra, dark blue dashed circles highlight the presence of amorphous DRN in the PVA/DRN ASD spectra, while purple dashed lines and circles highlight the differences among the PVA−PPSu/DRN ASDs and PMs).

Figure 11 presents the ATR-FTIR spectra of the raw materials, the neat amorphous API (prepared by melt-quench cooling), the binary API ASDs and the respective PMs (prepared by thoroughly mixing the PVA-PPSu SD matrices with the respective DRN crystalline drug in a mortar and pestle). In regard to the neat crystalline DRN, the recorded ATR-FTIR spectrum showed all characteristic FTIR peaks of the form I crystals at 3184 cm^−1^ due to sulfonamide NH stretching vibrations, 2956 cm^−1^ due to the aromatic C-H stretching vibrations, 1626 cm^−1^ due to C=O stretching vibrations and 1329 and 1144 cm^−1^ due to SO_2_ stretching. As for the differences between the crystalline and the amorphous API, the latter showed several noticeable shifts in the regions of 1700 to 1500 cm^−1^ and 1350 to 1110 cm^−1^, corresponding to the C=O and -SO_2_ stretching regions, respectively. Looking at the recorded binary (PVA/DRN) and ternary (PVA-PPSu/DRN) ATR-FTIR spectra for the PMs, it seems that in all cases the collected spectra were the sum of the individual crystalline API and the respective matrix-carrier. This finding suggests that no chemical interactions were formed between the API and the matrix-carriers during the preparation of the PMs. These results also verify that the mixing of the API with the carrier(s) did not induce any API’s amorphization since no DRN amorphous ATR-FTIR peaks were recorded. Similarly, in the case of the binary ASDs using only the polymer (PVA) as a matrix-carrier, the obtained spectra were mostly the sum of the two separate components, i.e., the crystalline DRN and PVA. However, looking more closely in the C=O stretching region of the ASDs and the respective PMs (1700 to 1500 cm^−1^), it is obvious that some portion of the API was transformed into its amorphous form, since the characteristic peaks of the amorphous DRN were also recorded in the obtained spectra. No other shifts were recorded in the obtained ASD spectra as compared to the respective PMs. Hence, it can be said that the preparation of DRN ASDs using only PVA as the matrix-carrier (i.e., without the addition of the plasticizer) at low working temperatures (170 °C) results in the partial amorphization of the API without the involvement of any molecular interactions between the two components.

On the contrary, in the case of the ternary ASDs (i.e., PVA-PPSu/DRN), the collected ATR-FTIR spectra were clearly the sum of the respective matrix-carrier and the amorphous API, since all characteristic amorphous DRN peaks were recorded. This indicates that the API was amorphously dispersed within the polymeric matrix when the PPSu polyester was added into the system. Furthermore, it is important to highlight that compared to the pure amorphous DRN, there is a significant shift of the DRN amorphous ATR-FTIR peak recorded at 1333 cm^−1^ (the shift is from 1333 cm^−1^ corresponding to the neat amorphous API to 1310 cm^−1^ corresponding to the amorphous API dispersed within the PVA-PPSu matrix-carrier), while there is also an additional significant broadening of the peak recorded at 1250 cm^−1^ (both changes are marked with purple dashed lines in Figure 10). Keeping in mind that the obtained ASD spectra also show a significant shift in the PVA-PPSu -C=O stretching peak (from 1726 to 1720 cm^−1^), it seems that the addition of the plasticizer results in the formation of significant molecular interactions between the API molecules and the matrix-carrier components. Although the intermolecular interactions play a crucial role in the preparation and performance of any ASD system, their formation is not always assured. As a previously published study [47] shows, when PPSu was evaluated as a matrix-carrier in the preparation of raloxifene HCL ASDs, the formation of molecular interactions between the components was not accomplishable and, consequently, the API was recrystallized.

#### 3.2.5. Evaluation of Supersaturated Dissolution Studies

**Figure 12:**
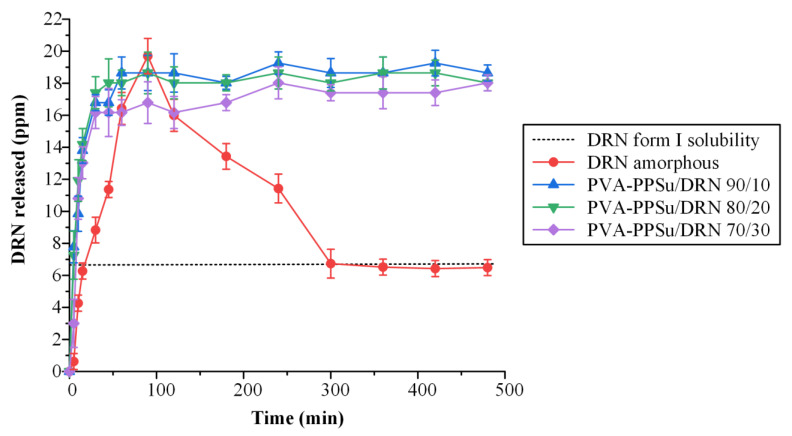
In vitro dissolution profiles under nonsink conditions of the neat amorphous DRN and all prepared ASDs (the dashed line represents the saturation solubility of DRN form I crystals).

Within the concept of any orally administered ASD system, one of its primary goals is to be able to increase API’s bioavailability (BA) in vivo. In this framework and based on the recent findings regarding the importance of in vivo drug supersaturation and precipitation, a well-designed ASD system should be able (amongst other features such as maintaining the API’s amorphous stability during storage) to preserve the drug’s high kinetic solubility by assuring continuous in vivo supersaturation. In this way, the rapid in situ drug nucleation, and hence recrystallization, which leads to the drug’s BA restriction, is prohibited [48,49,50,51]. Figure 12 shows the in vitro dissolution profiles of the ASDs conducted under nonsink conditions (SI = 0.15) as compared to the profile of the neat amorphous API and drug’s crystalline (form I) saturation solubility.

In regard to the neat amorphous API, results showed an initial increase in release and then a sharp decrease due to the rapid build-up of DRN supersaturation that led to the recrystallization of the API. This behavior is a typical behavior for amorphous drugs following a spring-like supersaturation kinetics profile in the absence of a precipitation inhibitor, where the initial high degree of supersaturation leads to drug nucleation and recrystallization. On the contrary, all prepared PVA-PPSu ASDs showed a rapid increase in API’s dissolution, as compared to the pure amorphous API, before building up and maintaining its supersaturation. ASDs of DRN prepared with the aid of Soluplus (SOL, an amphiphilic polyvinyl caprolactam–polyvinyl acetate–polyethylene glycol (PCL-PVAc-PEG) graft copolymer) showed a similar dissolution profile in pH 1.2, presenting an initial rapid increase in release, within the first 15 min, until a plateau was reached [52]. Based on the aforementioned findings, it seems that the evaluated PVA-PPSu polymeric matrices were able to inhibit the solution-mediated recrystallization of the API during dissolution, working hence as precipitation inhibitors. This finding agrees with similar previous studies showing that PVA is highly effective at inhibiting precipitation of itraconazole in aqueous media when evaluated as matrix-carrier in the preparation of drug ASDs [14]. It is strongly assumed that this inhibition effect is due to the previously reported interactions between drug and matrix-carrier components. Furthermore, the amphiphilic character of PVA, which provides both hydrophobic (acetate groups) and hydrophilic (hydroxyl groups) properties, plays an important role in stabilizing the supersaturated state. Additionally, it is important to note that the use of the plasticizer did not interfere with the precipitation inhibition effect of the polymer, hence indicating its suitability as a proper additive (i.e., plasticizer) in the preparation of PVA-based drug ASDs.

In addition, the superiority of the PVA-PPSu/DRN ASDs was also verified by calculating the area under the curve (AUC_(0->t)_) of the obtained supersaturation dissolution profiles, with results (summarized in Table 3) showing an almost 4-fold increase in the AUC_(0->t)_ of all ASDs as compared to crystalline API.

Finally, let us note that the in vitro delivery characteristics of DRN from the prepared ASDs (not only the supersaturation phenomena) may be highly dependent on the formation of molecular interactions during solubilization (i.e., within the dissolution medium) where self-assembly structures may be present, which can also be explained via -NH and -OH interactions.

#### 3.2.6. Storage Stability Results

**Figure 13:**
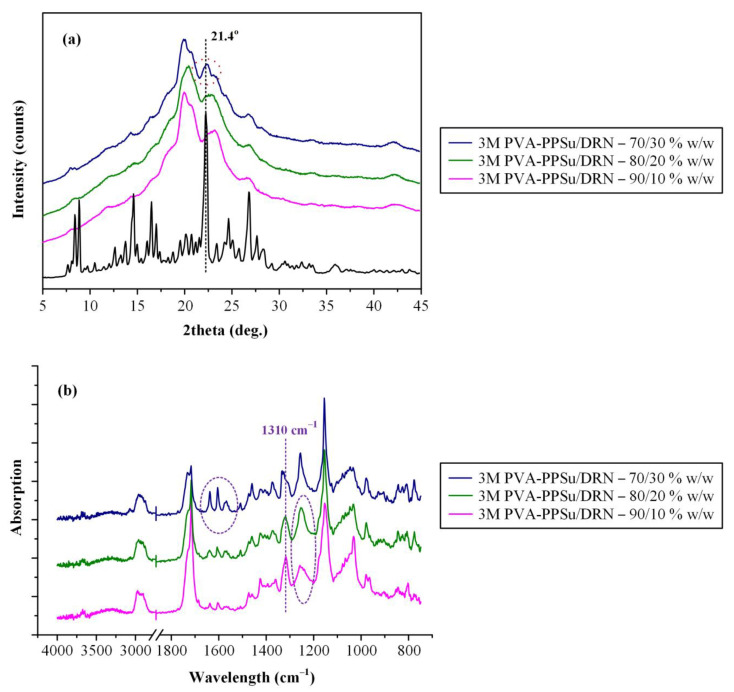
pXRD diffraction patterns (**a**) and ATR−FTIR spectra (**b**) of DRN-loaded PVA−PPsu ASDs after 3 months (3M) of storage.

In the final stage of the present study, the stability of the PVA-PPSu/DRN ASDs was evaluated after three months (3M) of storage using pXRD. Based on the obtained diffraction patterns shown in Figure 13a, it seems that DRN was stable (i.e., remained amorphous) only in the ASDs containing 10 and 20% wt. of API, while at higher drug concentrations (i.e., 30 wt.%), a peak at 21.4° was recorded, attributed to the presence of crystalline DRN. This instability can be ascribed to the presence of water molecules introduced into the system during storage, which can interact with the hydrophilic polymer chains, leading to the phase separation of the API and the polymeric matrix and consequently to API’s recrystallization, especially in the cases when high contents of the drugs are used [20,53,54,55].

Finally, ATR-FTIR spectroscopy was used in order to evaluate possible changes in the intermolecular interactions formed between the API and the polymeric matrix during storage. Results in Figure 13b showed that in the case of ASDs with low API content (i.e., up to 20 wt.%), the ATR-FTIR spectra collected after storage were the sum of the respective matrix-carrier (PVA-PPSu) and the amorphous API, indicating DRN remained amorphously dispersed within the prepared systems after 3M of storage. Additionally, in these cases, the intermolecular interactions between the API and the PVA-PPSu matrix-carrier observed in zero-time, i.e., immediately after preparation, were maintained also after storage (signified by the ATR-FTIR peak shift of amorphous DRN at 1310 cm^−1^ and the corresponding peak broadening at 1250 cm^−1^). On the contrary, in the case of the ASDs prepared using high drug loadings (i.e., 30 wt.%), the collected spectra after 3M of storage showed several peaks corresponding to the crystalline DRN form I (especially in the region of C=O stretching), indicating that a portion of the API recrystallized during storage. The spectra changes corresponding to the intermolecular interactions of the drug and the polymeric matrix-carrier seen in the zero–time ASDs were vanquished after 3M of storage.

## 4. Conclusions

The present study showed that the use of PPSu as a plasticizer may significantly improve the processability of PVA during fusion-based formulation processes, especially when there is a need for low thermal processing temperatures. This is extremely important when compounds (such as APIs or other additives) with low melting temperature or with poor thermal stability profile are being handled. With the utilization of PPSu, the processing temperature for the preparation of amorphous formulations was significantly reduced (for DRN this was at 170 °C) and the prepared ASDs showed good storage stability (at least in concentrations up to 20 wt.% of API) and sustained supersaturation. Therefore, based on the results of the present study, new application opportunities are emerging for formulation scientists working with PVA and ASDs, since some of the most significant drawbacks related to PVA’s thermal processing may be successfully alleviated.

## Figures and Tables

**Table 1:** Melting onset temperature (T_m,onset_), glass transition temperature (T_g_) and enthalpy of fusion (ΔH_f_) results based on the obtained DSC thermograms for the neat PVA/PPSu matrices.

Samples	DSC-Based Thermal Events						
	1st Heating Scan				2nd Heating Scan		
	PPSu		PVA				
	T_m,onset_ (°C)	ΔH_f_ (J/g)	T_m,onset_ (°C)	ΔH_f_ (J/g)	T_g_ (°C)	T_m,onset_ ** (°C)	ΔH_f_ (J/g)
PVA	-	-	171.1	42.5	68.4	140.1	30.8
PPSu	45.3	51.9	-	-	*	-	-
PVA/PPSu 90/10—3 min	-	-	149.5	41.4	67.2	135.2	28.9
PVA/PPSu 80/20—3 min	36.9	9.8	123.8	39.7	63.8	132.1	38.5
PVA/PPSu 70/30—3 min	42.1	10.2	112.7	28.7	63.3	125.0	21.3
PVA/PPSu 90/10—10 min	-	-	119.3	23.4	67.4	128.8	14.7
PVA/PPSu 80/20—10 min	-	-	114.2	18.0	64.4	98.3	19.8
PVA/PPSu 70/30—10 min	36.0	6.9	113.7	14.6	62.4	89.8	17.9

* T_g_ of the pure PPSu = −40.0 °C from [31]. ** Corresponds to the melting onset of PVA.

**Table 2:** Melting onset temperature (T_m,onset_), glass transition temperature (T_g_) and enthalpy of fusion (ΔH_f_) results based on the obtained DSC thermograms for the PVA/DVR and PVA-PPSu/DRN ASDs.

Samples	DSC-Based Thermal Events								
	1st Heating Scan						2nd Heating Scan		
	PPSu		PVA		DRN				
	T_m,onset_ (°C)	ΔH_f_ (J/g)	T_m,onset_ (°C)	ΔH_f_ (J/g)	T_m,onset_ (°C)	ΔH_f_ (J/g)	T_g_ (°C)	T_m,onset_ * (°C)	ΔH_f_ (J/g)
DRN	-	-	-	-	141.6	92.0	49.5	-	-
PVA/DRN 90/10	-	-	#	#	141.9	#	65.8	152.5	21.6
PVA/DRN 80/20	-	-	#	#	142.0	#	65.5	140.9	19.3
PVA/DRN 70/30	-	-	#	#	142.6	#	64.7	137.6	17.9
PVA-PPSu/DRN 90/10	38.4	8.4	133.5	23.1	-	-	55.7	142.5	16.7
PVA-PPSu/DRN 80/20	26.0	9.5	130.9	21.8	-	-	54.8	130.7	12.6
PVA-PPSu/DRN 70/30	24.9	11.7	129.5	15.3	-	-	54.5	119.5	9.9

* Corresponds to the melting onset of PVA in the sample. # T_m,onset_ and ΔH_f_ for PVA and ΔH_f_ for DRN cannot be determined since their DSC melting peaks overlap.

**Table 3:** Summary in vitro dissolution estimated AUC_(0 -> t)_ for the amorphous DRN and the ASDs, along with the estimated mean AUC_(0 -> t)_ ratio (i.e., AUC_(0 -> t)_ (sample)/AUC_(0 -> t)_ (DRN form I)).

Sample ID	AUC_(0 -> t)_ (ppm min × 10^3^) (Mean ± SD)	AUC_(0 -> t)_ Ratio (Mean)
DRN form I crystals	2.21 ± 0.05	1.00
DRN amorphous	4.94 ± 0.21	2.24
PVA-PPSu/DRN 90/10	8.74 ± 0.28	3.96
PVA-PPSu/DRN 80/20	8.60 ± 0.36	3.90
PVA-PPSu/DRN 70/30	8.04 ± 0.40	3.64

## Data Availability

Data are contained within the article.
